# In vitro and in silico scolicidal effect of sanguinarine on the hydatid cyst protoscoleces

**DOI:** 10.1371/journal.pone.0290947

**Published:** 2023-10-25

**Authors:** Elham Hassanzadeh, Shahram Khademvatan, Behzad Jafari, Abbas Jafari, Elham Yousefi

**Affiliations:** 1 Cellular and Molecular Research Center, Cellular and Molecular Medicine Institute & Department of Medical Parasitology and Mycology, Urmia University of Medical Sciences, Urmia, Iran; 2 Department of Medicinal Chemistry, School of Pharmacy Urmia University of Medical Sciences, Urmia, Iran; The Islamia University of Bahawalpur Pakistan, PAKISTAN

## Abstract

We aimed to investigate the scolicidal effects of sanguinarine on hydatid cyst protoscoleces (PSCs) in vitro and in silico. Different targets were docked into the active sites of sanguinarine. Molecular docking processes and visualization of interactions were performed using AutoDock Vina and Discovery Studio Visualizer. Binding energy was calculated and compared (kcal/mol). PSCs were aspirated from the hydatid cysts and washed. The sediments of PSCs were then exposed to various concentrations (50, 25, 12, 6, 3, and 1 μg/mL) of sanguinarine. The viability test was finally evaluated by the Trypan blue solution 4%. Levels of malondialdehyde (MDA), superoxide dismutase (SOD), glutathione (GSH), glutathione peroxidase (GPX), and catalase were analyzed to assess the level of oxidative stress-treated PSCs. Caspase-3 activity rate was determined to evaluate cell apoptosis in treated PSCs. Among the receptors, *acetylcholinesterase* was identified as the excellent target, with Vina score of -11.8. Sanguinarine showed high scolicidal effects after 12, 24, and 48 h. Also, in the first hour of exposure to the drug, caspase-3 activity and MDA level significantly increased, but the levels of GSH and GPx had a significant reduction after 12, 24, and 48 h (*P* < 0.05). The findings of this study revealed that sanguinarine have potent scolicidal effects in vitro and in silico and could be considered an opportunity for the introduction of a novel and safe therapeutic agent for the treatment of cystic echinococcosis. However, supplementary studies will be desired to prove the current findings by examining sanguinarine in a clinical setting.

## Introduction

*Cystic echinococcosis* (CE) is a known disease caused by *Echinococcus (E*.*) granulosus*, a parasite found in the small intestine of dogs [1]. The parasite’s life cycle often occurs between definitive hosts such as dogs and other canines and livestock. Humans, as an intermediate host, are infected by eating parasite eggs, excreted along with the feces of the final host. The parasite grows in its larval form as hydatid cysts in various organs (heart, brain, bone, kidneys, and particularly liver and lungs) of the intermediate host [2, 3]. In humans, CE can cause clinical manifestations, ranging from asymptomatic to life-threatening symptoms, eventually leading to death [4]. CE also poses a health issue around the globe, especially in developing countries such as Iran [5, 6]. Therefore, rapid diagnosis and effective therapy before and after surgery can help control and treat the disease [3, 7]. Clinical management strategies for live cysts include surgery, percutaneous methods, chemotherapy, and silent cysts "watch and wait" [8]. However, using protoscolicidal substances in s surgery and also percutaneous methods has limitations due to severe side effects such as bile duct fibrosis [9]. Surgery as an excellent treatment options carries risks such as anaphylactic shock and secondary recurrence as a result of protoscolece (PSCs) leakage [10]. In virtue of these difficulties, a suitable protoscolicidal agent without side effects is needed during surgery.

The use of a chemo-informatics method in search for drug design has become an inseparable research approach as it is highly effective, efficient, rapid, and economical. Molecular docking acts a very significant role in the rational design of drugs. This technique allows predicting the affinity and activity of a drug candidate molecule with the protein as a receptor [11].

*E*. *granulosus* is well adapted to its intermediate host and resists oxidative stress caused by the host defense system or drug therapy. This parasite also develops antioxidant defense mechanisms, including a system of enzymatic (e.g. superoxide dismutase [SOD], glutathione peroxidase [GPx], and catalase) and non-enzymatic (e.g. GSH) antioxidants [12]. Oxidative stress causes damage to essential biomolecules and cells and is followed by lipid peroxidation, which gives rise to an increase in malondialdehyde (MDA) [13, 14]. Evaluation of biomarkers of oxidative stress such as MDA, GSH, and enzymatic antioxidants is important in detecting the level of oxidative stress [12].

Many efforts have been made to discover a scolicidal substance from different sources, including plants and microorganisms, as well as from the extracts of marine bioactive compounds [15–17]. Sanguinarine is a potential drug candidate and an alkaloid compound isolated from the roots of *Sanguinaria canadensis* and the seeds of *Argemone mexicana*, *Poppy fumaria*, *Bocconia frutescens*, *Chelidonium majus*, and *Macleaya cordata*. It posesses biological (antibacterial, antiviral, antifungal, anti-inflammatory, and antitumor) features [18, 19], as well as antihelmintic properties in vivo against *Dactylogyrus intermedius* [20]. Sanguinarine has also shown an antiparasitic effect against *Ichthyophthirius multifiliis* and antischistosomal activity in vivo and in vitro [21, 22]. Its potential anticancer attributes have recently been reported, as well [23]. Sanguinarine induces apoptosis through multiple mechanisms, comprising NF-κB activation, mitochondrial damage, and cell cycle arrest [23]. Unlike necrosis, apoptosis is an essential regulated process of cell death. One of the vital mediators of apoptosis is caspases, among which, caspase-3 is a frequently activated death protease [24].

Considering common treatment problems in hydatidosis, this study aimed to investigate the scolicidal effect of sanguinarine on hydatid cyst using in silico methods and viability tests and to assess oxidative stress biomarkers. Examining potential drugs with high scolicidal effects and evaluating drug targets in *E*. *granulosus* could contribute to the identification of novel and highly effective agents for the treatment of CE with minimal or no side effects and also hydatid cyst surgery without risk.

## Material and methods

This study was approved by the Ethical Committee of Urmia University of Medical Sciences, Urmia, Iran at (http://ris.umsu.ac.ir/&ethics.research.ac.ir), reference number (**IR.UMSU.REC.1400.446**).

### Protein target prediction by PharmMapper

The three-dimensional (3D) structure of sanguinarine was submitted to the PharmMapper (http://www.lilab-ecust.cn/pharmmapper/). PharmMapper is a freely available web server designed to identify potential target candidates for given small molecules (drugs, natural products, or other newly discovered compounds with unidentified binding targets) using a pharmacophore mapping approach. The results downloaded from PharmMapper included all the information for protein-compound docking with ligand in the Protein Data Bank.

### BLAST search with *E*. granulosus sequence and homology modeling

Sequence of protein targets was retrieved from the UniProt server (https://www.uniprot.org/). The alignment of the target sequences in *E*. *granulosus* was carried out by using the basic local alignment search tool (BLAST). This tool lists database entries (DNA or protein sequences) ordered according to their alignment scores, E-value (statistical measure), and position in the query sequence. The sequence alignments were used to generate homology models with SWISS-MODEL for higher-ranked BLAST results. Also, the structure of sanguinarine was sketched, and energy was minimized using built-in modules of HyperChem 8.0.

### Three-dimensional structure validation

Quality of the generated model was assessed by PROCHECK, a program that is based on Ramachandran plots for structure verification, figures out the stereochemical quality of the model [25].

### Molecular docking

AutoDock 4.2.6 software was used to prepare input files. Interactions and docking position of target proteins with sanguinarine and docking calculations were carried out using AutoDock Vina 4.2, an open-source software for molecular docking. In AutoDock Vina, the binding energy parameters indicate which target has a more reasonable interaction with the ligand; the lower binding affinity, the more satisfactory the ligand-receptor interaction.

### Protein-ligand interactions

The molecular interaction between the ligand and the target receptor in the binding pocket can be checked and viewed in two dimensions by the aid of Discovery studio visualizer 2016 software. This two-dimensional (2D) visualization of receptor-ligand interactions display molecular connections, hydrophobicity, hydrogen bonding, bond distances, amino acid interaction, aromaticity, and so forth. The above-mentioned software Discovery studio visualizer 2016 software was used to achieve 2D and 3D interaction diagrams for the selected target-ligand complex.

### Isolation of protoscoleces (PSCs)

Hydatid cysts were collected from the livers and lungs of infected sheep in an industrial slaughterhouse in Urmia, the Northwestern Iran, and transferred to the parasitology laboratory of Urmia University of Medical Science, Urmia, Iran. The surface of the infected cysts was sterilized with alchohol 75%, and the contents of the cysts were aspirated by syringes and transferred to sterile tubes. The sediment of PSCs was washed three times with phosphate-buffered saline (PBS; pH 7.2).

### Viability test of PSCs

Sanguinarine purchased from Sigma-Aldrich(CAS no. 4752-86-7). In vitro protoscolicidal effects of sanguinarine (at doses 1, 3, 6, 12, 25, and 50 μg/mL were examined for 15 min, 30 min, 60 min, 12 h, 24 h, and 48 h. The drug dose was used from low to high to find the lowest dose with the best scolicidal effect. After the PSCs were treated with different concentrations of sanguinarine at the mentioned times, the viability rate of the PSCs was evaluated by the Trypan blue test. Briefly, 50 μL of 0.4% Trypan blue solution was added to the treated PSCs, smeared on a glass slide, covered with a coverglass and evaluated under a light microscope. There after, 100 μL of different concentrations of sanguinarine was exposed to 1,000 PSCs six times; all samples had viability >90% at the time of experiments. PSC viability was then determined in each experiment. Viable PSCs remained colorless and transparent and showed flame cell activity. About 150 protoscolecses were counted each time, to determine the initial percentages [26]. Finally, the ratio of dead to live PSCs was counted on an entire surface of the slide with a light microscope. PBS and Savlon were considered as negative and positive controls, respectively. All experiments were carried out in triplicate [27].

### Analysis of oxidative stress biomarkers and caspase-3 activity

#### Caspase-3 activity assay

Caspase-3 activity was measured in samples by colorimetric assay kit according to the manufacturer’s protocol (ZellBio GmbH, Germany, Cat No. RK01037). The principle of this assay is based on the ability of caspase-3 to hydrolyze Ac-DEVD-pNA and release para-nitro aniline (pNA). Cleavage of the pNA produced a yellow color, which is measured by an ELISA reader at 405 nm. The amount of yellow color produced upon cleavage is proportional to the amount of caspase activity present in the sample.

#### MDA assay

The MDA level is a marker of lipid peroxidation in samples and was measured by a chemical colorimetrical assay kit (ZellBio GmbH, Germany, Cat No. E-BC-K028-M-50)) according to the manufacturer’s protocol. MDA can react with thiobarbituric acid in acid medium to generate a pink-colored complex that can be measured colorimetrically at 535 nm, named as thiobarbituric acid reactive substances.

#### GSH assay

GSH is known as non-enzymatic antioxidants involved in detoxification of xenobiotics. Measurement of GSH level was performed with a chemical colorimetric assay kit (ZellBio GmbH Germany, Cat No. ZX-44100-96) using colorimetrically method at 412 nm.

#### GPx activity

The activity of GPx was measured by a chemical colorimetrical assay kits (ZellBio GmbH, Germany, Cat No. RK09254-96) at the wavelength of 412 nm, according to the manufacturer’s procedure. The unit of GPx activity was determined as the quantity of sample and was used to catalyze 1 μmole of GSH to GSSG in 1 min.

#### SOD activity

SOD activity was measured using a calorimetrically enzymatic assay kit(ZellBio GmbH, Germany, Cat No. E-BC-K020-M-48) at the wavelength of 420 nm. One unit of SOD activity was defined as the amount of the sample and was used to catalyze 1 μmole of O2- to H_2_O_2_ and O^2^ in 1 min.

#### Catalase activity assay

The catalase activity was determined by a colorimetrical assay kit (ZellBio GmbH Germany, Cat No. E-BC-F006-96) at 405 nm as recommended by the manufacturer. In this study, the CAT activity unit was considered as the amount of the sample that catalyzed the decomposition of 1 μmole of H_2_O_2_ to water and O_2_ in 1 min.

PSCs were treated with different concentrations (50, 25, 12, 6 μg/mL) of sanguinarine for 1h, 12 h, 24 h, and 48 h for SOD, MDA, GSH, GPX, catalase, and caspase-3 activity tests. The reason for selecting these time periods is that this drug leads to cell apoptosis through the activation of the caspase cascade, and it needs a long time to be effective. Sediments of PSCs were washed three times, homogenized in 200 μl of lysis buffer and centrifuged at 12,000 ×g at 4°C for 10 min. The supernatant was separated, stored at -80°C and used to determine the levels of SOD, MDA, GSH, GPX, and catalase by kits, according to the manufacturer’s procedure. Also, the value of caspase-3 activity in the supernatant was measured using the caspase-3 Activity Assay Kit (ZellBio GmbH, Germany). PBS and Savlon were considered as negative and positive controls, respectively. All experiments were performed in triplicate.

### Statistical analysis

The results of the scolicidal effect of sanguinarine were reported as mean ± standard deviation. Statistical analysis of data was conducted using GraphPad Prism 9 and SPSS 22.0 software. One-way ANOVA analysis with post-hoc (Tukey) was employed to assess the variations among tested groups in most biological studies. *P* < 0.05 was considered statistically significant.

## Results

### Validation of the models and molecular docking analysis

From the alignment of the target sequences in *E*. *granulosus*, 100 targets were selected as template proteins based on query cover, maximum identity, and statistical E-value (Table 1). Homology modeling was carried out for higher-ranked BLAST results. The quality of models was evaluated by Global Model Quality Estimation (GMQE), QMEANDisCo global, and sequence identity (Table 2). For each target, the highest quality models were generated. The structural integrity of the 3D models were validated by PROCHECK (Ramachandran plot). Ramachandran plot (Fig 4) of the modeled *acetylcholinesterase*, *cytochrome c oxidase* (*COX*), glycerol-3-phosphate dehydrogenase (GPDH), and uridine-cytidine kinase (UCK) represented as 81.9% (835 aa), 91.6% (404 aa), 91,6% (557 aa), and 89.9% (223 aa) of the total residues in most favored regions and 14.1% (144 aa), 7.5% (33 aa), 8.4% (51 aa), and 8.5% (21 aa) in additionally allowed regions, respectively, implying the high quality of the models. Finally, the docking of 300 structures with sanguinarine was performed. Docking analysis was conducted by evaluating the binding affinity score. Table 3 shows the binding energy of the best 10 out of 300 target receptors. A more negative binding energy indicates stronger binding or higher favorable orientation between our target proteins and sanguinarine.

**Table 1:** Putative *Echinococcus granulosus* drug targets.

score	name	blast_best	max score	total score	query cover	E value	per. Iden	Acc. Lent	max score	total score
1	Acetylcholinesterase_03_ligand	XP_024348463.1	312	312	85%	3.00E-97	34.83%	704	312	312
2	cytochrome_oxidase_subunit_1_partial_03_ligand	QIC34371.1	247	247	72%	7.00E-74	31.49%	536	247	247
3	Glycerol3phosphate_dehydrogenase_NAD_cytop_02_ligand	KAH9281035.1	362	362	97%	9.00E-125	53.33%	362	362	362
4	uridine_cytidine_kinase_1_1_02_ligand	CDS21471.1	83.2	83.2	91%	2.00E-18	31.28%	570	83.2	83.2
5	Kelchlike_protein_3_02_ligand	KAH9281829.1	198	283	90%	2.00E-54	26.94%	829	198	283
6	uridine_cytidine_kinase_1_1_03_ligand	CDS21471.1	83.2	83.2	91%	2.00E-18	31.28%	570	83.2	83.2
7	Glycerol3phosphate_dehydrogenase_NAD_cytop_03_ligand	KAH9281035.1	362	362	97%	9.00E-125	53.33%	362	362	362
8	Acetylcholinesterase_01_ligand	XP_024348463.1	312	312	85%	3.00E-97	34.83%	704	312	312
9	Ribosephosphate_pyrophosphokinase_1_01_ligand	KAH9285566.1	553	553	100%	0	81.76%	360	553	553
10	Ribosephosphate_pyrophosphokinase_1_02_ligand	KAH9285566.1	553	553	100%	0	81.76%	360	553	553

**Table 2:** Property and quality of homology modeling.

score	name	GMQE	QMEANDisCo Global	Template	Seq Identity
1	Acetylcholinesterase_03_ligand	0.53	0.60 ± 0.05	6h14.1.A	41.89%
2	cytochrome_oxidase_subunit_1_partial_03_ligand	0.7	0.69 ± 0.05	5z62.1.A	49.60%
3	Glycerol3phosphate_dehydrogenase_NAD_cytop_02_ligand	0.82	0.79 ± 0.05	1x0x.2.A	58.62%
4	uridine_cytidine_kinase_1_1_02_ligand	0.32	0.73 ± 0.05	1upf.1.A	43.72%
5	Kelchlike_protein_3_02_ligand	0.31	0.79 ± 0.05	5nkp.2.A	56.79%
6	uridine_cytidine_kinase_1_1_03_ligand	0.33	0.73 ± 0.05	1upu.1.A	43.72%
7	Glycerol3phosphate_dehydrogenase_NAD_cytop_03_ligand	0.8	0.79 ± 0.05	2pla.1.A	52.91%
8	Acetylcholinesterase_01_ligand	0.55	0.64 ± 0.05	6ary.1.A	41.14%
9	Ribosephosphate_pyrophosphokinase_1_01_ligand	0.82	0.87 ± 0.05	2h07.1.E	81.45%
10	Ribosephosphate_pyrophosphokinase_1_02_ligand	0.8	0.87 ± 0.05	2h07.1.A	81.45%

**Table 3:** Docking results of the best 10 receptors with sanguinarine (showing the lowest binding energy).

score	name	Binding affinity (Kcal.mol-1)	UniProt
**1**	Acetylcholinesterase_03_ligand	-11.8	P21836
**2**	cytochrome_oxidase_subunit_1_partial_03_ligand	-11.5	P0ABI8
**3**	Glycerol3phosphate_dehydrogenase_NAD_cytop_02_ligand	-11.5	Q8N335
**4**	uridine_cytidine_kinase_1_1_02_ligand	-11.2	Q72J35
**5**	Kelchlike_protein_3_02_ligand	-11.1	Q9H2C0
**6**	uridine_cytidine_kinase_1_1_03_ligand	-11.1	Q72J35
**7**	Glycerol3phosphate_dehydrogenase_NAD_cytop_03_ligand	-11	Q8N335
**8**	Acetylcholinesterase_01_ligand	-10.9	P21836
**9**	Ribosephosphate_pyrophosphokinase_1_01_ligand	-10.8	P60891
**10**	Ribosephosphate_pyrophosphokinase_1_02_ligand	-10.8	P60891

### Interaction of sanguinarine with best target receptors

**Fig 1:**
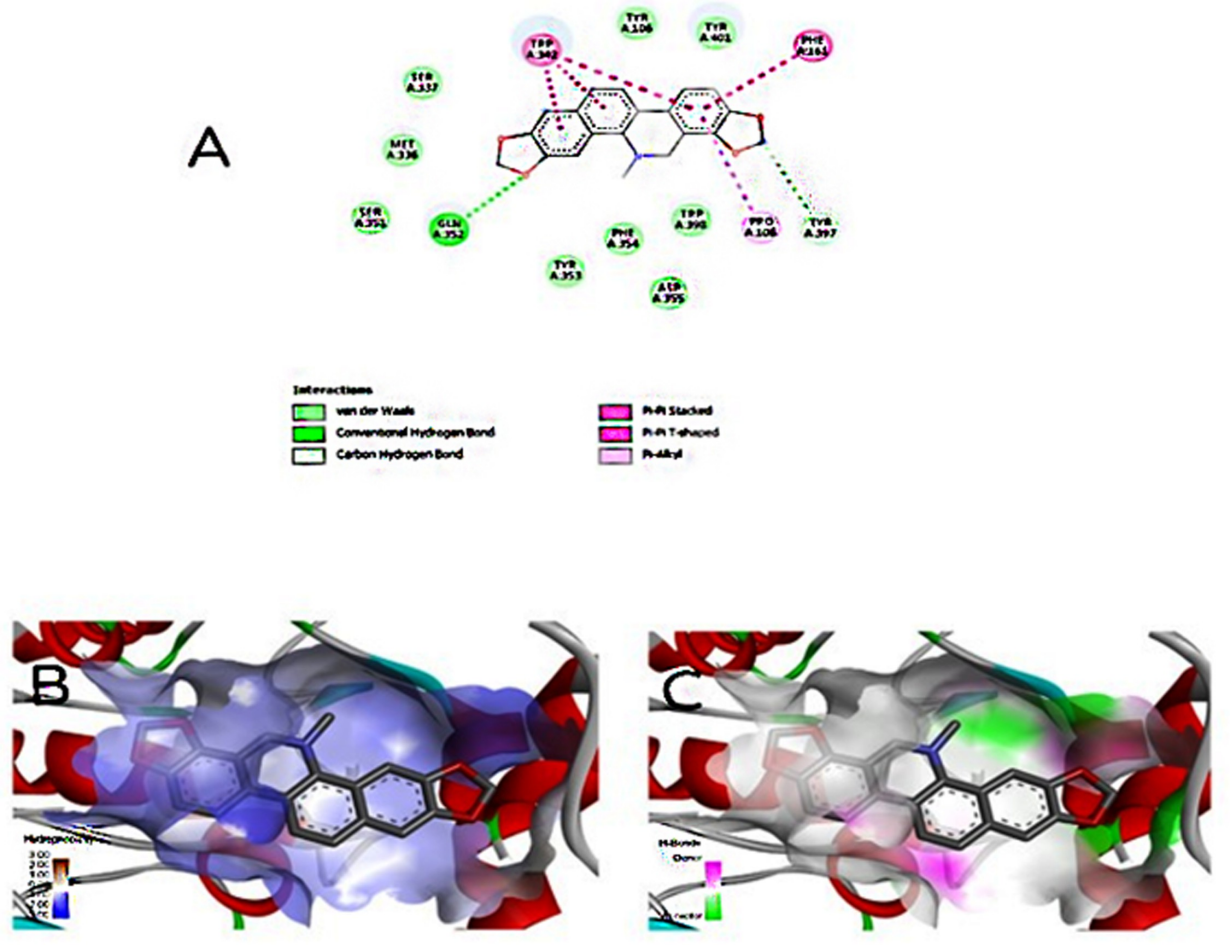
(A) Interaction of the ligand sanguinarine with the acetylcholinesterase in the pocket (sanguinarine has formed two hydrogen bonds with the amino acids GLN A:352 and TYR A:397). It has hydrophobic interactions with TRP A:342, PHE A:161, PRO A:108. (B and C) Hydrophobic interaction and hydrogen bond of sanguinarine with acetylcholinesterase in 3D, respectively.

**Fig 2:**
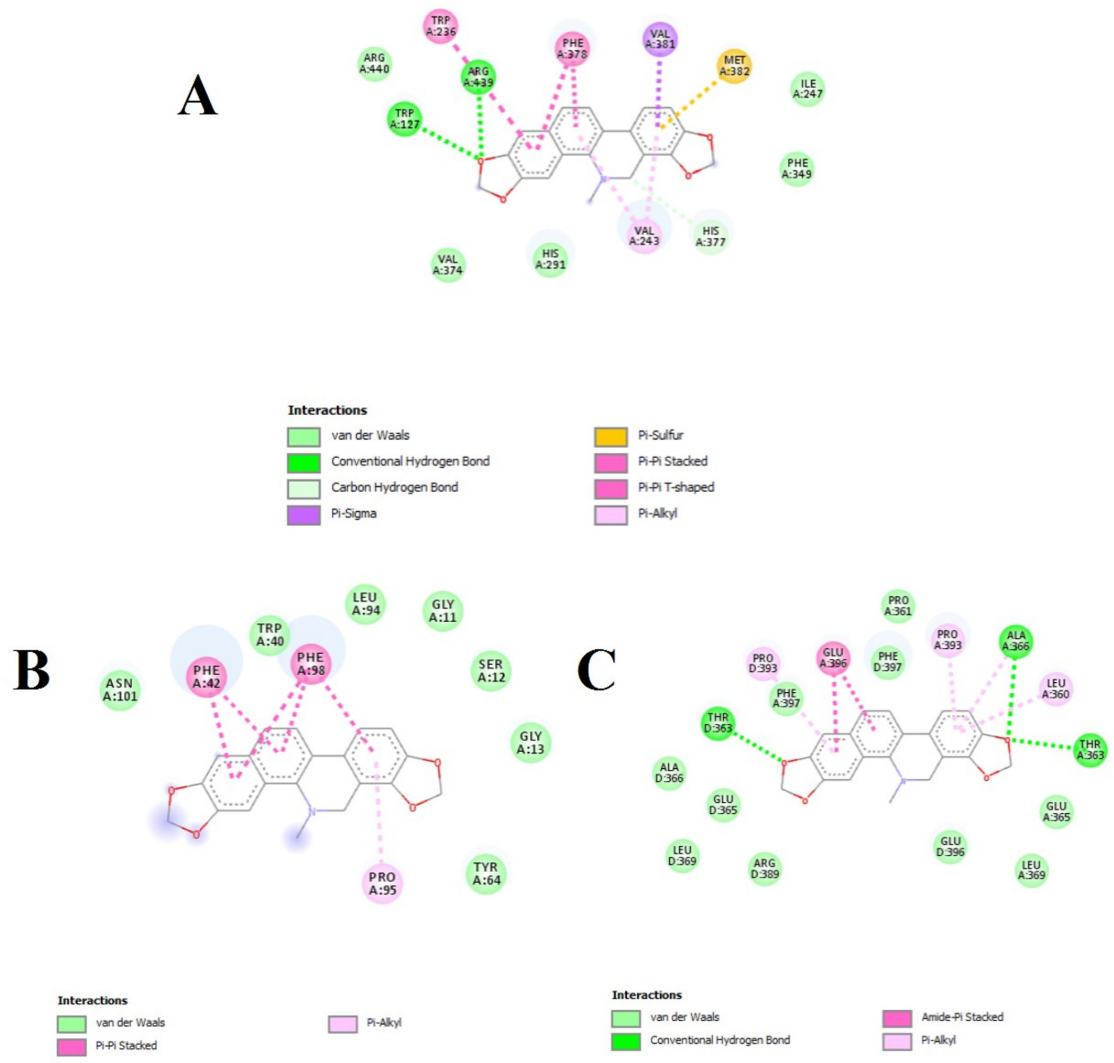
Interaction of the ligand sanguinarine with cytochrome oxidase, glycerol 3-phosphate dehydrogenase, and uridine-cytidine kinase in the pocket (A, B, and C, respectively). Sanguinarine in the catalytic site of cytochrome oxidase has three hydrogen bonds with amino acids ARG A:439, TRP A:127, HIS A:377 and has five hydrophobic interactions with PHE A:378, TRP A:236, and VLA A:243. In the catalytic site of Glycerol3phosphate_dehydrogenase, it has six hydrophobic interactions with amino acids PHE A:98, PHE A:42 and PRO A:95. Also in the catalytic site of uridine_cytidine_kinase, it has three hydrogen bonds with amino acids THR A:363, ALA A:366 and THR D:363 and 6 hydrophobic interactions with PRO A:393, PRO D:393, GLU A:396, and LEU A:360.

**Fig 3:**
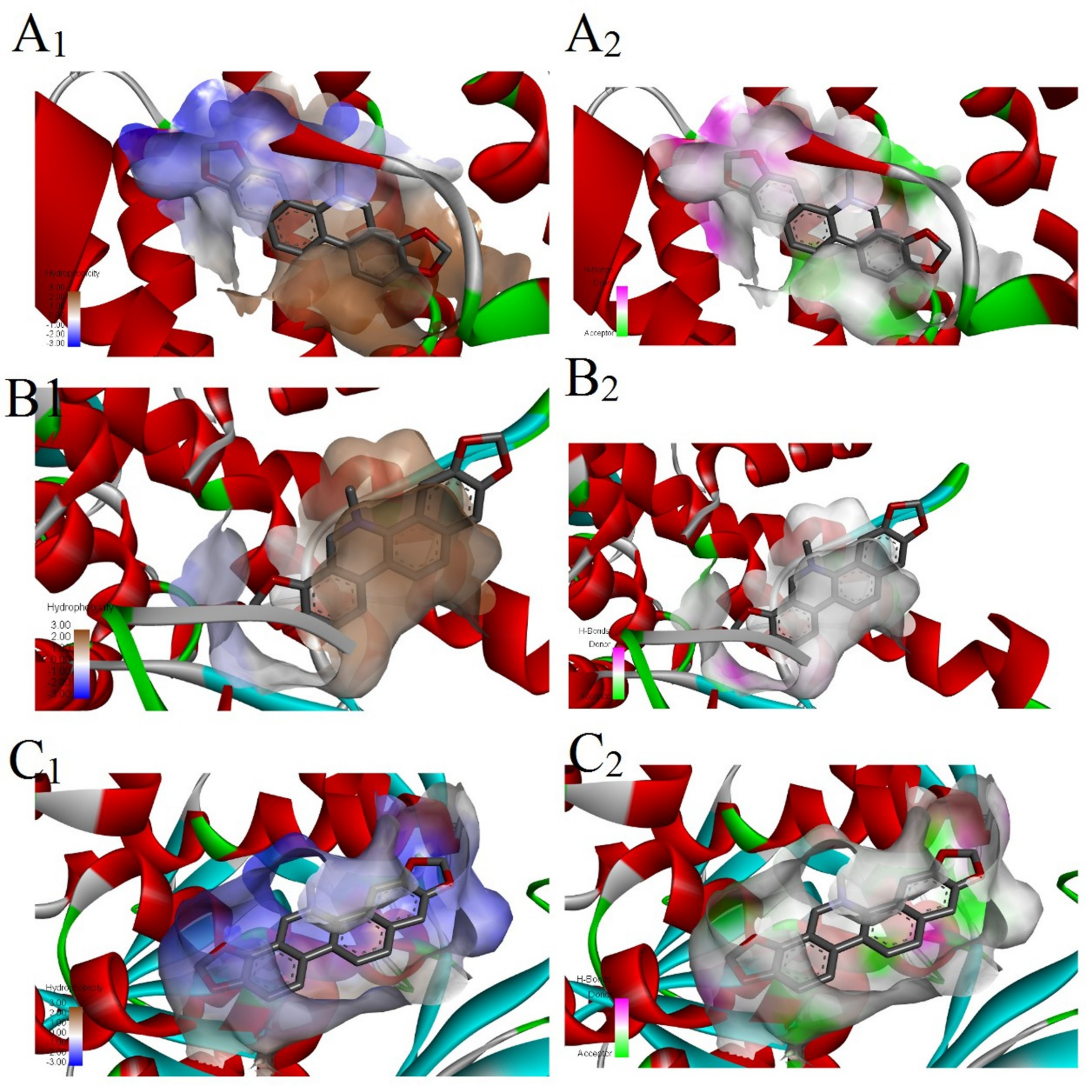
Hydrophobic interaction (pictures with subscript 1) and hydrogen bond (pictures with subscript 2) of sanguinarine with cytochrome oxidase, glycerol 3-phosphate dehydrogenase, and uridine-cytidine kinase in 3D (A, B, and C, respectively).

Amino acids at the catalytic site of acetylcholinesterase were identified from the 2D interaction diagram. Sanguinarine consists of two hydrogen bonds with the amino acids GLN A:352 and TYR A:397 and has hydrophobic interactions with TRP A:342, PHE A:161, and PRO A:108. Also, the bond between acetylcholinesterase and sanguinarine of hydrophobic type and hydrogen bond was obtained in 3D with the help of Discover Studio software (Fig 1). Acetylcholinesterase is a potent receptor with the highest Vina score (-11.8 kcal/mol) and the lowest binding energy. In the catalytic site of *COX*, sanguinarine has three hydrogen bonds with amino acids ARG A:439, TRP A:127, and HIS A:377 and has five hydrophobic interactions with PHE A:378, TRP A:236, and VLA A:243. In the catalytic site of GPDH, sanguinarine has six hydrophobic interactions with amino acids PHE A:98, PHE A:42, and PRO A:95. However, in the catalytic site of UCK, it has three hydrogen bonds with amino acids THR A:363, ALA A:366, and THR D:363 and six hydrophobic interactions with PRO A:393, PRO D:393, GLU A:396, and LEU A:360 (Fig 2). The hydrophobic interactions and hydrogen bonds between sanguinarine and *COX*, GPDH, and UCK were determined in three dimensions (Fig 3). *COX*, GPDH, and UCKwere the second, third, and fourth best targets for sanguinarine, with Vina scores of -11.5, -11.5, and -11.2, respectively.

### In vitro scolicidal effects of sanguinarine

**Fig 4:**
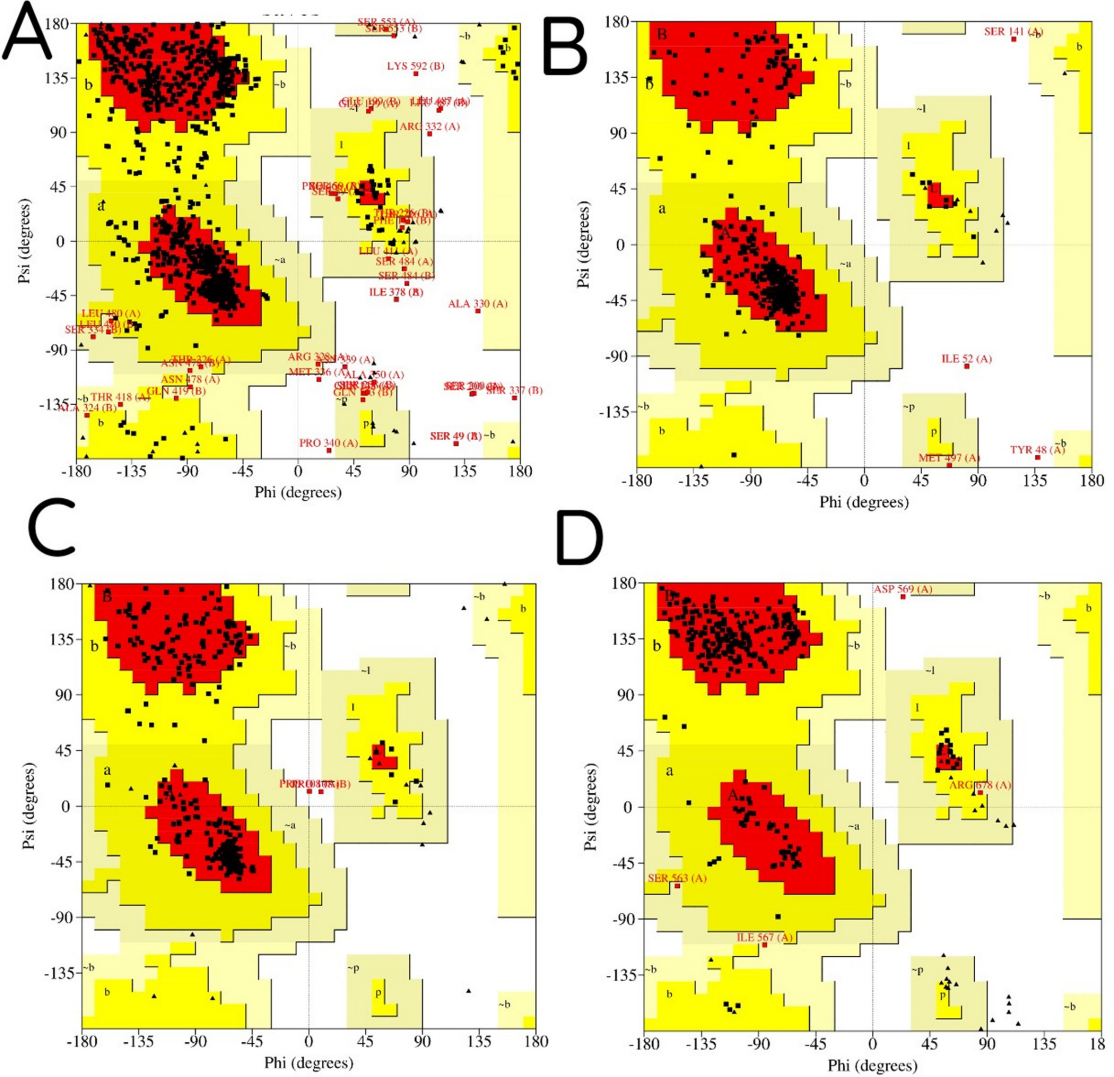
Stereochemical analysis of acetylcholinesterase, cytochrome oxidase, glycerol-3-phosphate dehydrogenase and uridine-cytidine kinase (A, B, C, and D, respectively). The red regions represent the most favorable area of residues; the yellow regions is additionally allowed; and generously allowed residues in the light-yellow region. the Ramachandran plot denotes 96%, 99,1%, 100%, and 98.4% of residues falling in the allowed region, respectively.

The viability rate of hydatid cysts PSCs after exposure to different concentrations (1, 3, 6, 12, 25, and 50 μg/ml) of sanguinarine, positive control, and negative control in the desired time periods (15 min, 30 min, 60 min, 12 h, 24 h, and 48 h) is represented in Fig 4. The significantly highest scolicidal effects of sanguinarine were observed after 12, 24, and 48 h of exposure (*P* < 0.05). The mortality rate was found to be 14% in the negative control group (PBS) after 48-h exposure to 50 μg/ml of sanguinarine, while this rate was 100% for the positive control group (Savlon) exposed to sanguinarine (50 μg/ml) in the same time period.

### Caspase-3 activity

**Fig 5:**
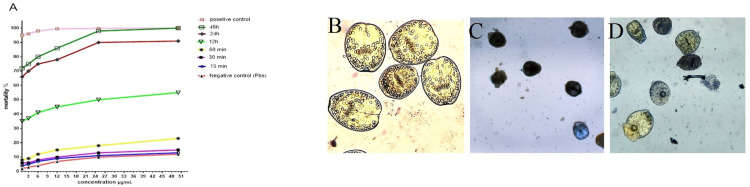
(A) Scolicidal effects of sanguinarine and positive control and negative control in different concentrations and times; (B) live protoscoleces, observed as colorless and transparent; (C and D) dead cells, observed as blue due to dye’s penetration.

Results indicated the increased levels of caspase-3 activity after treatment with sanguinarine. No significant changes were observed in the level of caspase-3 activity in the first hour of exposure to the drug, but after 12 and 24 hours of exposure, there was a significant elevation in caspase-3 activity in all the groups. Also, after 48 hours of exposure to the sanguinarine, an increase was detected in the caspase-3 activity level in all the groups, but this elevation was not significantly different from 12 and 24 h of exposure (*P* < 0.05; Fig 5A).

### MDA levels

Based on the obtained results, the levels of MDA increased after treatment with sanguinarine. No significant alterations were found in the MDA level in the first hour of exposure to the drug; however, after 12 and 24 h of exposure to sanguinarine, there was a significant elevation in the amount of MDA in all the groups. In addition, after 48 hours of exposure to the drug, an increase was observed in the rate of MDA in all the groups, but this increase was not significantly different from 12 and 24 h of exposure (*P* < 0.05; Fig 5B).

### GSH and GPx levels

**Fig 6:**
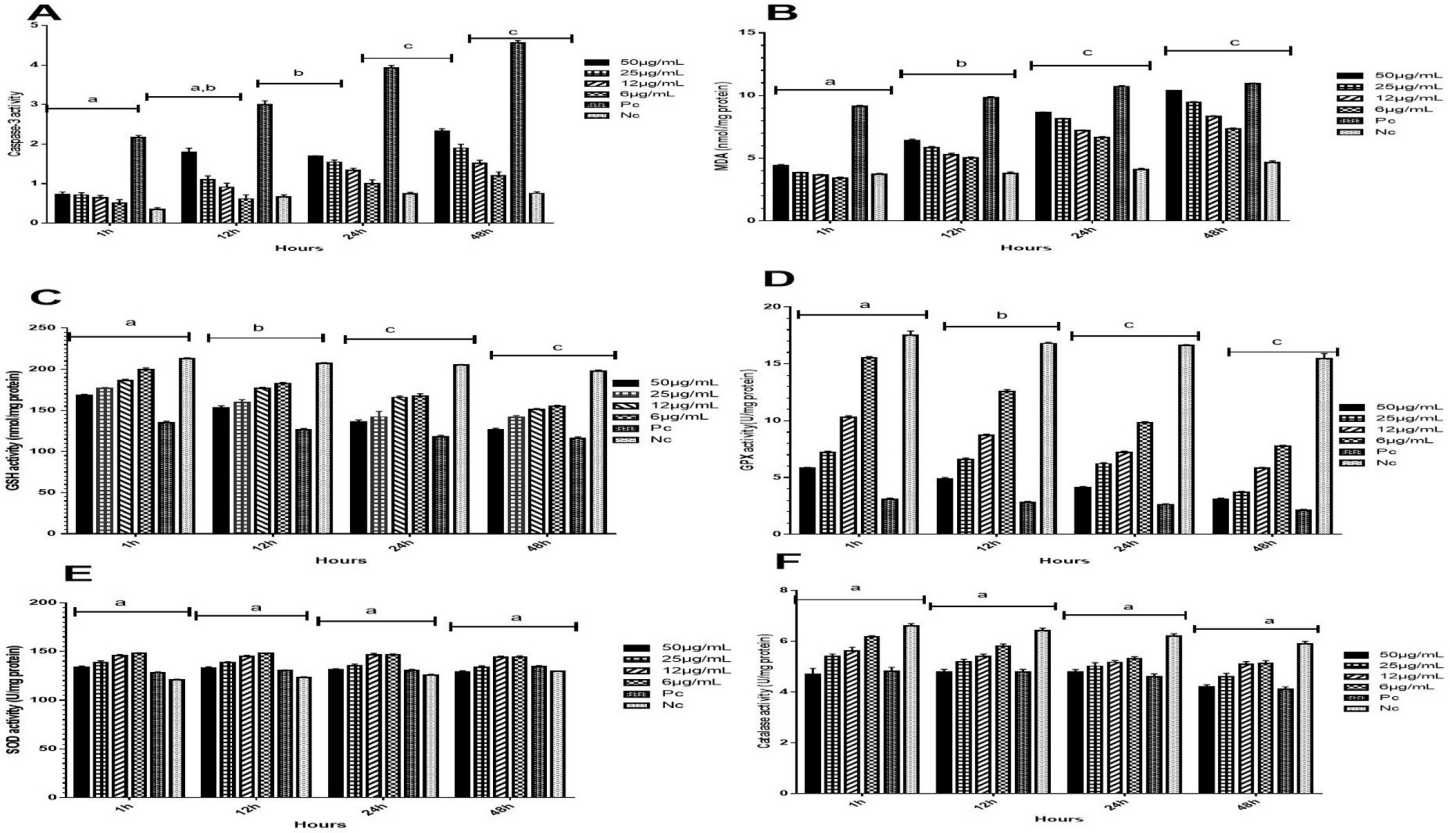
Caspase-3 activity, MDA, GSH, GPX, SOD, and catalase assay in protoscoleces exposed to different concentrations (50, 25, 12, and 6 μg/ml) of sanguinarine and positive control in the time periods of 1, 12, 24, and 48 h (A, B, C, D, E, and F, respectively). Results were presented as the mean ± SD, n = 3. The groups with different superscript letters have significant differences *(P < 0*.*05)*.

The results demonstrated a reduction in the levels of GSH and GPX after treatment with sanguinarine. Their levels also showed no significant alterations in the first hour of exposure to the drug, but after 12 and 24 h of exposure, there was a significant decrease in the levels of GSH and GPx in all the groups. In addition, after 48 hours of exposure to the sanguinarine drug, a decrease was found in the GSH and GPx levels in all the groups. However, this decrease was not significantly different from 12 hand 24 hours of exposure (*P* < 0.05; Fig 6C and 6D, respectively).

### SOD and catalase activity levels

According to the obtained results, levels of SOD and catalase activity decreased after treatment with sanguinarine but this reduction did not change significantly with passing time between all the groups (*P* < 0.05; Fig 6E and 6F, respectively).

## Discussion

Due to the lack of and the side events of scolicidal agents for the treatment of hydatid cyst [28, 29], the presence of an effective scolicidal drug with low side effects is essential. Herbal medicines, owing to low cost, easy access, and few adverse reactions, have recently attracted a great interest among researchers to provide alternative herbal treatments [2]. In this study, we investigated the scolicidal effects of sanguinarine in vitro by Trypan blue staining and in silico by homology modeling tools (virtualization modeling), virtual screening, and docking analysis.

Herein, a docking study was performed using AutoDock Vina 4.2 to understand the interactions of sanguinarine with different receptor targets and to explore their binding mode. To this end, the energy binding of ligand (sanguinarine) and receptors (targets) was calculated and compared (kcal/mol). *Acetylcholinesterase* with Vina score of -11.8 was identified as the superior receptor target, followed by *COX*, GPDH, and UCK, with Vina scores of -11.5, -11.5, and -11.2, respectively. Similar investigations have also used in silico methods to investigate the effect of various ligands (drugs) on the drug target in Leishmania parasite [30, 31]. In another similar study, the activity of *acetylcholinesterase* was examined in *E*. *granulosus*, and organophosphates were tested as specific inhibitors of *acetylcholinesterase*. That study revealed that PSCs of *E*. *granulosus* have cholinesterase activities, and these activities could be considered as possible drug targets in chemotherapy [32]. Our results exhibited that *acetylcholinesterase* is the best drug target with the lowest binding affinity in *E*. *granulosis*. An earlier study evaluated PSCs in the encystation process using high-throughput RNA sequencing and showed that antioxidant enzymes, including *COX* and *GPx*, had a high expression rate. According to the COG database annotation, *COX*, an upregulated differentially expressed gene, has the molecular function of binding to heme [33]. Previous reports have indicated that *COX* dysfunction is associated with increased mitochondrial reactive oxygen species (ROS) production, as well as apoptosis. Through external environmental pressures, *COX* in PSCs may play key roles in the antioxidant responses during the encystation process [34, 35]. In our study, *COX* ranked the second among the best drug target for sanguinarine in *E*. *granulosis*. In a recent survey, hydrogen peroxide (H^2^O^2^) was used to induce oxidative stress in PSCs in order to identify molecular pathways and antioxidant defenses during H_2_O_2_ exposure. Among proteins responding to H_2_O_2_, enzymes related to oxido-reductase activity (estradiol 17-beta-dehydrogenase and protein disulfide isomerase) and glycerol metabolism (GPDH) were found [12]. Some of these enzymes such as GPDH are targets for chemotherapy in cancer and are related to protozoan drug resistance [36].

The docking results of our study displayed that GPDH ranked in the third place among the best targets for sanguinarine in *E*. *granulosis*. Using idTarget web server, Liu and colleagues also identified drug targets of amino alcohols with effects on Echinococcus species. The 3D structures of the drug targets were then built following the BLAST sequence analysis and homology modeling. After further screening by molecular docking, they detected glycogen phosphorylase, as a potential drug target for amino alcohols [37].

*Acetylcholinesterase*, *COX*, GPDH, and UCK were explored in this study as the best potential drug targets for sanguinarine, respectively. Ramdhani and Kusuma evaluated the mechanism of interaction between eugenol compounds from *Eugenia caryophyllus* against receptors associated with antibacterial activity. In this regard, they used three target receptors with antibacterial activity, comprising penicillin-binding protein 3, N-myristoyltransferase, and cytochrome P450 14 alpha-sterol demethylase. The binding activity data achieved from molecular docking showed that the eugenol compound, at three receptors, had less antibacterial effect than each native ligand [38]. In our study, the docking results of binding affinity displayed the reasonable interaction and strong binding affinity of sanguinarine with the identified targets.

In the current study, to investigate the scolicidal effect of sanguinarine, we exposed PSCs to different concentrations (1, 3, 6, 12, 25, and 50 μg/ml) of the drug in the desired time periods (15 min, 30 min, 60 min, 12 h, 24 h, and 48 h). The viability test showed a high mortality rate of PSC after its exposure to sanguinarine for 12, 24, and 48 h (*P* < 0.05). Two similar studies investigated and proved the larvicidal effects of sanguinarian on *Trichinella spiralis* as well as its antihelmintic property [20, 39]. Using the viability test, researchers have confirmed the potential scolicidal effects of various medicinal compounds, such as isofuranodiene, α-bisabolol, farnesol, Albendazole sulfoxides-loaded poly(lactic-co-glycolic acid)-poly(ethylene glycol), and Foeniculum vulgare Mill [40–42]. In our study, four effective drug concentrations (50, 25, 12, and 6 μg/ml) were exposed to PSCs for 60 min, 12, 24, and 48h. After homogenization, the supernatant was analyzed to measure SOD, MDA, GSH, GPX, catalase, and caspase-3 activity levels. GSH and GPX decreased more significantly after 12 h, but caspase-3 activity significantly elevated in the same time period. This increase behavior is likely due to the feature of sanguinarine in activating caspase-3 activity cascade and inducing apoptosis. Following lipid peroxidation, MDA level also increased significantly after 12 h (*P* < 0.05).

Sanguinarine has shown various activities such as disrupting microtubules and inducing apoptosis [18]. It has also demonstrated antimicrobial effects, as well as anticancer activity through inducing apoptosis and antiproliferative effect on tumor cells. The antitumor property of sanguinarine probably arises from its proapoptotic and growth-inhibiting properties of tumor cells and its anti-angiogenic and anti-invasive attributes. Sanguinarine induces apoptosis in tumor cells through multiple mechanisms, such as NF-κB activation, mitochondrial damage, and cell cycle arrest. Apoptosis induced by this compound is associated with the reduction of Bcl-2, the increase of BOX proteins, and the production of ROS [23]. In our study, sanguinarine has shown apoptotic effect on PSC.

While the mechanism of scolicidal activity is unknown, it is speculated that essential oils can cause membrane depolarization and membrane potential reduction, there by decreasing pH gradient. The reduction of pH gradients affects not only the proton pump activity but also the adenosine triphosphate pool. In addition, the essential oils changes the fluidity of mitochondrial membrane, which results in the leakage of radicals, cytochrome c, and calcium, ultimately leading to the cell death by apoptosis [43, 44]. Some compounds, such as *Nigella sativa*, can inhibit histone deacetylase enzymes and DNA synthesis, while some other, such as *Myrtus communis*, can increase the activity of caspase-3 and caspase-9 [45, 46]. The apoptotic effect of praziquantel and dexamethasone on PSC have been proven using terminal deoxynucleotidyl transferase dUTP nick end labeling (TUNEL) assay and caspase-3 enzymatic activity [47, 48]. In our study, sanguinarine led to an increased level of caspase-3 activity, followed by PSC apoptosis.

Antioxidant enzymes present in *E*. *granulosus* are involved in antioxidant stress reaction and removal of ROS, which protects the parasite from oxidative damage [12]. Therefore, it is expected that the property of an antioxidant substance declines following the oxidative stress and its exposure to harmful materials [14, 49]. In the study of Xu et al., GSH and SOD were measured to assess the scolicidal effect of lithocholic acid. Their results showed a significant reduction in the levels of these oxidative stress biomarkers (*P* < 0.05) [50]. Li et al. evaluated the scolicidal effect of arsenic trioxide and observed a significant reduction in the SOD level (*P* < 0.05) [51].

Due to the potential of sanguinarine in activating the caspase cascade and inducing apoptosis, the highest rate of caspase-3 activity and MDA, and the lowest level of GSH and antioxidant enzymes were observed after 48, 24, and 12 h. After 1 hour of exposure to the drug, no significant change was found, probably because the production of enzymes and proteins involved in the process of apoptosis and biomarkers of oxidative stress had not reached a detectable level. Our finding revealed that the scolicidal effects of sanguinarine are mostly time-dependent.

## Conclusion

The present study for the first time evaluated the scolicidal effect of sanguinarine in vitro and in silico. Various drug targets were identified in *E*. *granulosis* and ranked based on the binding energy. This research confirmed the strong scolicidal effect and the dose- and time-dependent scolicidal activity of sanguinarine. However, these results should be investigated with molecular and experimental studies before being introduced as a suitable drug in the treatment of hydatidosis. Likewise, the ability of live PSCs to generate hydatid cysts needs further evaluation in vivo as sanguinarine-exposed PSCs may be alive but have lost the ability of hydatid cyst formation.

## Supporting information

S1 Table Mortality rate. (DOCX)

S2 Table Caspase3 activity in different time pointes. (DOCX)

S3 Table MDA in different time pointes. (DOCX)

S4 Table GSH in different time pointes. (DOCX)

S5 Table Catalase in different time pointes. (DOCX)

S6 Table SOD in different time pointes. (DOCX)

S7 Table GPX in different time pointes. (DOCX)

## References

[1] Guidelines for treatment of cystic and alveolar echinococcosis in humans. WHO Informal Working Group on Echinococcosis. Bull World Health Organ. 1996;74(3):231–42. 8789923 PMC2486920

[2] Kohansal MH, Nourian A, Rahimi MT, Daryani A, Spotin A, Ahmadpour E. Natural products applied against hydatid cyst protoscolices: A review of past to present. Acta Trop. 2017 Dec;176:385–94. doi: 10.1016/j.actatropica.2017.09.013 28935552

[3] Pakala T, Molina M, Wu GY. Hepatic Echinococcal Cysts: A Review. J Clin Transl Hepatol. 2016 Mar 28;4(1):39–46. doi: 10.14218/JCTH.2015.00036 27047771 PMC4807142

[4] Brunetti E, Kern P, Vuitton DA. Expert consensus for the diagnosis and treatment of cystic and alveolar echinococcosis in humans. Acta Trop. 2010 Apr;114(1):1–16. doi: 10.1016/j.actatropica.2009.11.001 19931502

[5] Khademvatan S, Majidiani H, Foroutan M, Hazrati Tappeh K, Aryamand S, Khalkhali HR. Echinococcus granulosus genotypes in Iran: a systematic review. J Helminthol. 2019 Mar;93(2):131–8. doi: 10.1017/S0022149X18000275 29606162

[6] Khalkhali HR, Foroutan M, Khademvatan S, Majidiani H, Aryamand S, Khezri P, et al. Prevalence of cystic echinococcosis in Iran: a systematic review and meta-analysis. J Helminthol. 2018 May;92(3):260–8. doi: 10.1017/S0022149X17000463 28589871

[7] Venukumar R. Clinical presentation of hydatid cyst of liver: descriptive study. International Surgery Journal. 2016;4(1):214–6. 10.18203/2349-2902.isj20164395

[8] McManus DP, Zhang W, Li J, Bartley PB. Echinococcosis. Lancet. 2003 Oct 18;362(9392):1295–304. doi: 10.1016/S0140-6736(03)14573-4 14575976

[9] Sahin M, Eryilmaz R, Bulbuloglu E. The effect of scolicidal agents on liver and biliary tree (experimental study). J Invest Surg. 2004 Nov-Dec;17(6):323–6. doi: 10.1080/08941930490524363 15764499

[10] Junghanss T, da Silva AM, Horton J, Chiodini PL, Brunetti E. Clinical management of cystic echinococcosis: state of the art, problems, and perspectives. Am J Trop Med Hyg. 2008 Sep;79(3):301–11. 18784219

[11] Cheng T, Li Q, Zhou Z, Wang Y, Bryant SH. Structure-based virtual screening for drug discovery: a problem-centric review. Aaps j. 2012 Mar;14(1):133–41. doi: 10.1208/s12248-012-9322-0 22281989 PMC3282008

[12] Cancela M, Paes JA, Moura H, Barr JR, Zaha A, Ferreira HB. Unraveling oxidative stress response in the cestode parasite Echinococcus granulosus. Sci Rep. 2019 Nov 4;9(1):15876. doi: 10.1038/s41598-019-52456-3 31685918 PMC6828748

[13] Sri Iswari R, Dafip M, Purwantoyo E. Malondialdehyde (MDA) Production in Atherosclerosis Supplemented with Steamed Tomato. Pak J Biol Sci. 2021 Jan;24(3):319–25. doi: 10.3923/pjbs.2021.319.325 34486316

[14] Ijaz MU, Tahir A, Samad A, Anwar H. Nobiletin ameliorates nonylphenol-induced testicular damage by improving biochemical, steroidogenic, hormonal, spermatogenic, apoptotic and histological profile. Hum Exp Toxicol. 2021 Mar;40(3):403–16. doi: 10.1177/0960327120950007 32815738

[15] Anthony JP, Fyfe L, Smith H. Plant active components ‐ a resource for antiparasitic agents? Trends Parasitol. 2005 Oct;21(10):462–8. doi: 10.1016/j.pt.2005.08.004 16099722

[16] Navvabi A, Homaei A, Khademvatan S, Khadem Ansari MH, Keshavarz M. In vitro study of the scolicidal effects of Echinometra mathaei spine and shell extracts on hydatid cyst protoscolices. Exp Parasitol. 2019 Aug;203:19–22. doi: 10.1016/j.exppara.2019.05.009 31153894

[17] Aryamand S, Khademvatan S, Hazrati Tappeh K, Heshmatian B, Jelodar A. In Vitro and in Vivo Scolicidal Activities of Holothuria leucospilota Extract and CeO2 Nanoparticles against Hydatid Cyst. Iran J Parasitol. 2019 Apr-Jun;14(2):269–79. 31543915 PMC6737367

[18] Mackraj I, Govender T, Gathiram P. Sanguinarine. Cardiovasc Ther. 2008 Spring;26(1):75–83. doi: 10.1111/j.1527-3466.2007.00037.x 18466423

[19] Caballero-George C, Vanderheyden PM, Apers S, Van den Heuvel H, Solis PN, Gupta MP, et al. Inhibitory activity on binding of specific ligands to the human angiotensin II AT1 and endothelin 1 ETA receptors: Bioactive benzo [c] phenanthridine alkaloids from the root of Bocconia frutescens. Planta medica. 2002;68(09):770–5. 10.1055/s-2002-34406 12357384

[20] Wang GX, Zhou Z, Jiang DX, Han J, Wang JF, Zhao LW, et al. In vivo anthelmintic activity of five alkaloids from Macleaya microcarpa (Maxim) Fedde against Dactylogyrus intermedius in Carassius auratus. Vet Parasitol. 2010 Aug 4;171(3–4):305–13. doi: 10.1016/j.vetpar.2010.03.032 20413222

[21] Yao JY, Shen JY, Li XL, Xu Y, Hao GJ, Pan XY, et al. Effect of sanguinarine from the leaves of Macleaya cordata against Ichthyophthirius multifiliis in grass carp (Ctenopharyngodon idella). Parasitol Res. 2010 Oct;107(5):1035–42. doi: 10.1007/s00436-010-1966-z 20625767

[22] Zhang SM, Coultas KA. Identification of plumbagin and sanguinarine as effective chemotherapeutic agents for treatment of schistosomiasis. Int J Parasitol Drugs Drug Resist. 2013 Dec;3:28–34. doi: 10.1016/j.ijpddr.2012.12.001 23641325 PMC3638872

[23] Gaziano R, Moroni G, Buè C, Miele MT, Sinibaldi-Vallebona P, Pica F. Antitumor effects of the benzophenanthridine alkaloid sanguinarine: Evidence and perspectives. World J Gastrointest Oncol. 2016 Jan 15;8(1):30–9. doi: 10.4251/wjgo.v8.i1.30 26798435 PMC4714144

[24] Xu X, Lai Y, Hua ZC. Apoptosis and apoptotic body: disease message and therapeutic target potentials. Biosci Rep. 2019 Jan 31;39(1). doi: 10.1042/BSR20180992 30530866 PMC6340950

[25] Laskowski RA, MacArthur MW, Moss DS, Thornton JM. PROCHECK: a program to check the stereochemical quality of protein structures. Journal of applied crystallography. 1993;26(2):283–91. 10.1107/S0021889892009944

[26] Sarvestani A, Karimian A, Mohammadi R, Cheraghipour K, Zivdri M, Nourmohammadi M, et al. Scolicidal effects of Cassia fistula and Urtica dioica extracts on protoscoleces of hydatid cysts. Journal of Parasitic Diseases. 2021;45(1):59–64. doi: 10.1007/s12639-020-01273-x 33746387 PMC7921229

[27] Besim H, Karayalcin K, Hamamci O, Güngör C, Korkmaz A. Scolicidal agents in hydatid cyst surgery. HPB surgery. 1998;10(6):347–51. doi: 10.1155/1998/78170 9515230 PMC2423906

[28] Bensghir M, Fjouji S, Bouhabba N, Ahtil R, Traore A, Azendour H, et al. Anaphylactic shock during hydatid cyst surgery. Saudi J Anaesth. 2012 Apr;6(2):161–4. doi: 10.4103/1658-354X.97031 22754444 PMC3385260

[29] Yılmaz F, Kaplan C, Naebi N. Anaphylaxis due to liver hydatid cyst during the operation. Cyprus J Med Sci. 2018;3:112–3. doi: 10.5152/cjms.2018.401

[30] Khademvatan S, Adibpour N, Eskandari A, Rezaee S, Hashemitabar M, Rahim F. In silico and in vitro comparative activity of novel experimental derivatives against Leishmania major and Leishmania infantum promastigotes. Exp Parasitol. 2013 Oct;135(2):208–16. doi: 10.1016/j.exppara.2013.07.004 23872452

[31] Khademvatan S, Eskandari K, Hazrati-Tappeh K, Rahim F, Foroutan M, Yousefi E, et al. In silico and in vitro comparative activity of green tea components against Leishmania infantum. J Glob Antimicrob Resist. 2019 Sep;18:187–94. doi: 10.1016/j.jgar.2019.02.008 30797085

[32] Giménez-Pardo C, Ros Moreno RM, De Armas-Serra C, Rodríguez-Caabeiro F. Presence of cholinesterases in Echinococcus granulosus protoscolices. Parasite. 2000 Mar;7(1):47–50. doi: 10.1051/parasite/2000071047 10743648

[33] Fan J, Wu H, Li K, Liu X, Tan Q, Cao W, et al. Transcriptomic Features of Echinococcus granulosus Protoscolex during the Encystation Process. Korean J Parasitol. 2020 Jun;58(3):287–99. doi: 10.3347/kjp.2020.58.3.287 32615742 PMC7338903

[34] Zalewska A, Ziembicka D, Żendzian-Piotrowska M, Maciejczyk M. The Impact of High-Fat Diet on Mitochondrial Function, Free Radical Production, and Nitrosative Stress in the Salivary Glands of Wistar Rats. Oxid Med Cell Longev. 2019;2019:2606120. doi: 10.1155/2019/2606120 31354904 PMC6637679

[35] Ozcan C, Li Z, Kim G, Jeevanandam V, Uriel N. Molecular Mechanism of the Association Between Atrial Fibrillation and Heart Failure Includes Energy Metabolic Dysregulation Due to Mitochondrial Dysfunction. J Card Fail. 2019 Nov;25(11):911–20. doi: 10.1016/j.cardfail.2019.08.005 31415862 PMC7144800

[36] Irie J, Murata M, Homma S. Glycerol-3-phosphate Dehydrogenase Inhibitors, Anacardic Acids, from Ginkgo biloba. Biosci Biotechnol Biochem. 1996 Jan;60(2):240–3. doi: 10.1271/bbb.60.240 27299399

[37] Liu C, Yin J, Hu W, Zhang H. Glycogen Phosphorylase: A Drug Target of Amino Alcohols in Echinococcus granulosus, Predicted by a Computer-Aided Method. Front Microbiol. 2020;11:557039. doi: 10.3389/fmicb.2020.557039 33329421 PMC7719768

[38] Ramdhani D, Kusuma SAF. Molecular docking method: antibacterial activity of eugenol from clove plant. World Journal of Pharmaceutical Research. 2022;11(2):19–28. doi: 10.20959/wjpr20222-22802

[39] Huang H, Yao J, Liu K, Yang W, Wang G, Shi C, et al. Sanguinarine has anthelmintic activity against the enteral and parenteral phases of trichinella infection in experimentally infected mice. Acta Trop. 2020 Jan;201:105226. doi: 10.1016/j.actatropica.2019.105226 31634454

[40] Youssefi MR, Nikpay A, Hassanpour N, Mirzapour A, Tabari PS, Pavela R, et al. In Vitro Scolicidal Activity of the Sesquiterpenes Isofuranodiene, α-Bisabolol and Farnesol on Echinococcus granulosus Protoscoleces. Molecules. 2020 Aug 7;25(16). 10.3390/molecules25163593 PMC7464821 32784679

[41] Naseri M, Akbarzadeh A, Spotin A, Akbari NA, Mahami-Oskouei M, Ahmadpour E. Scolicidal and apoptotic activities of albendazole sulfoxide and albendazole sulfoxide-loaded PLGA-PEG as a novel nanopolymeric particle against Echinococcus granulosus protoscoleces. Parasitol Res. 2016 Dec;115(12):4595–603. doi: 10.1007/s00436-016-5250-8 27623699

[42] Lashkarizadeh MR, Asgaripour K, Saedi Dezaki E, Fasihi Harandi M. Comparison of Scolicidal Effects of Amphotricin B, Silver Nanoparticles,_and Foeniculum vulgare Mill on Hydatid Cysts Protoscoleces. Iran J Parasitol. 2015 Apr-Jun;10(2):206–12. 26246818 PMC4522296

[43] Arunasree K. Anti-proliferative effects of carvacrol on a human metastatic breast cancer cell line, MDA-MB 231. Phytomedicine. 2010;17(8–9):581–8. doi: 10.1016/j.phymed.2009.12.008 20096548

[44] Yang Y, Yue Y, Runwei Y, Guolin Z. Cytotoxic, apoptotic and antioxidant activity of the essential oil of Amomum tsao-ko. Bioresour Technol. 2010 Jun;101(11):4205–11. doi: 10.1016/j.biortech.2009.12.131 20133123

[45] Sharifi-Rad J, Mnayer D, Tabanelli G, Stojanović-Radić ZZ, Sharifi-Rad M, Yousaf Z, et al. Plants of the genus Allium as antibacterial agents: From tradition to pharmacy. Cell Mol Biol (Noisy-le-grand). 2016 Aug 29;62(9):57–68. 10.14715/cmb/2016.62.9.10 27585263

[46] Shahnazi M, Azadmehr A, Jondabeh MD, Hajiaghaee R, Norian R, Aghaei H, et al. Evaluating the effect of Myrtus communis on programmed cell death in hydatid cyst protoscolices. Asian Pac J Trop Med. 2017 Nov;10(11):1072–6. doi: 10.1016/j.apjtm.2017.10.010 29203104

[47] De S, Pan D, Bera AK, Sreevatsava V, Bandyopadhyay S, Chaudhuri D, et al. In vitro assessment of praziquantel and a novel nanomaterial against protoscoleces of Echinococcus granulosus. J Helminthol. 2012 Mar;86(1):26–9. doi: 10.1017/S0022149X10000908 21281527

[48] Hu H, Kang J, Chen R, Mamuti W, Wu G, Yuan W. Drug-induced apoptosis of Echinococcus granulosus protoscoleces. Parasitol Res. 2011 Aug;109(2):453–9. doi: 10.1007/s00436-011-2276-9 21365454

[49] Peterson QP, Goode DR, West DC, Botham RC, Hergenrother PJ. Preparation of the caspase-3/7 substrate Ac-DEVD-pNA by solution-phase peptide synthesis. Nat Protoc. 2010 Feb;5(2):294–302. doi: 10.1038/nprot.2009.223 20134429 PMC2921128

[50] Xu Y, Qing W, Wang Z, Chen L, Wang L, Lv H, et al. In vitro protoscolicidal effects of lithocholic acid on protoscoleces of Echinococcus granulosus and its mechanism. Exp Parasitol. 2022 Aug;239:108280. doi: 10.1016/j.exppara.2022.108280 35594934

[51] Li J, Tang G, Qin W, Yang R, Ma R, Ma B, et al. Toxic effects of arsenic trioxide on Echinococcus granulosus protoscoleces through ROS production, and Ca2+-ER stress-dependent apoptosis. Acta Biochim Biophys Sin (Shanghai). 2018 Jun 1;50(6):579–85.vdoi: 10.1093/abbs/gmy041 29684096

