# Supplementary material for: In vitro and in silico scolicidal effect of sanguinarine on the hydatid cyst protoscoleces
Source: PLoS One. 2023 Oct 25;18(10):e0290947. doi: 10.1371/journal.pone.0290947 (PMC10599545; doi:10.1371/journal.pone.0290947)
Supplement: S1 Table — (DOCX) [file pone.0290947.s001.docx]

S1 Table. Mortality rate

| concentration drug | 15min | 30 min | 1h | 12h | 24h | 48h |
| --- | --- | --- | --- | --- | --- | --- |
| 1 μg/ml | **4±0/97** | **5±1/1** | **8±1/67** | **35±2/1** | **66±1/19** | **72±1/79** |
| 3 μg/ml | **5±1/08** | **6±0/87** | **9±0/77** | **37±1/75** | **70±1/21** | **75±0/89** |
| 6 μg/ml | **7±1/61** | **8±0/76** | **12±1/92** | **41±0/98** | **75±1/2** | **80±1/39** |
| 12 μg/ml | **9±0/56** | **10±1/17** | **15±0/82** | **45±1/2** | **78±0/79** | **86±1/19** |
| 25 μg/ml | **11±2/1** | **13±1/81** | **18±0/90** | **50±1/61** | **90±0/86** | **98±0/9** |
| 50 μg/ml | **13±0/92** | **15±0/98** | **23±1/35** | **55±0/79** | **91±1/45** | **100±0/0** |
| Positive control | **95± 0/9** | **95±0/3** | **96±0/7** | **100±0/0** | **100±0/0** | **100±0/0** |
| Negative control | 2±0/1 | 3±0/21 | 4±0/14 | 7±0/24 | 10±0/18 | 12±0/11 |
