# Supplementary material for: In vitro and in silico scolicidal effect of sanguinarine on the hydatid cyst protoscoleces
Source: PLoS One. 2023 Oct 25;18(10):e0290947. doi: 10.1371/journal.pone.0290947 (PMC10599545; doi:10.1371/journal.pone.0290947)

S2 Table. Caspase3 activity in different time pointes

| **Time**  **Concentration** | **1h** | **12h** | **24h** | **48h** |
| --- | --- | --- | --- | --- |
| **50 μg/ml** | 0.70±0/15 | 1.8±0.12 | 1.66±0/32 | 2.30±2/14 |
| **25 μg/ml** | 0.66±1/22 | 1.1±0.73 | 1.54±0/60 | 1.90±1/72 |
| **12 μg/ml** | 0.59±0/19 | 0.94±0.5 | 1.33±1/75 | 1.45±0/51 |
| **6 μg/ml** | 0.40±0/54 | 0.65±0.32 | 0.99±1/5 | 1.2±0/9 |


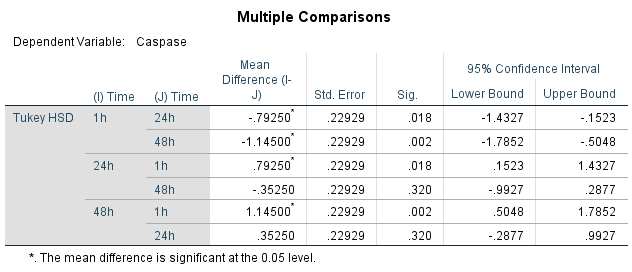

Supplement: S2 Table — (DOCX) [file pone.0290947.s002.docx]
