# Supplementary figures and images for: In vitro and in silico scolicidal effect of sanguinarine on the hydatid cyst protoscoleces
Source: PLoS One. 2023 Oct 25;18(10):e0290947. doi: 10.1371/journal.pone.0290947 (PMC10599545; doi:10.1371/journal.pone.0290947)

S3 Table. MDA in different time pointes

| **Time**  **Concentration** | **1h** | **12h** | **24h** | **48h** |
| --- | --- | --- | --- | --- |
| **50 μg/ml** | 4.40±0/19 | 6.41±0.14 | 8.60±0/48 | 10.39±1/44 |
| **25 μg/ml** | 3.79±1/38 | 5.8±0.21 | 8.11±1/20 | 9.57±0/82 |
| **12 μg/ml** | 3.69±0/91 | 5.3±0.32 | 7.16±0/57 | 8.31±0/87 |
| **6 μg/ml** | 3.41±0/75 | 5.01±0.56 | 6.61±2/1 | 7.24±1/5 |


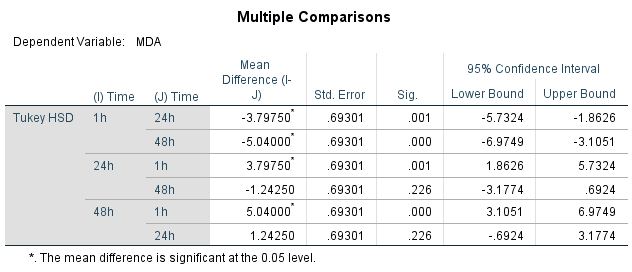

Supplement: S3 Table — (DOCX) [file pone.0290947.s003.docx]

S4 Table. GSH in different time pointes

| **Time**  **Concentration** | **1h** | **12h** | **24h** | **48h** |
| --- | --- | --- | --- | --- |
| **50 μg/ml** | 168.01±0/9 | 153±0.25 | 136.16±0/2 | 127.17±1/3 |
| **25 μg/ml** | 177.13±0/85 | 159±0.81 | 142.01±1.5 | 141.87±0/55 |
| **12 μg/ml** | 187.69±o/24 | 176±0.15 | 166.42±0/74 | 151.29±0/12 |
| **6 μg/ml** | 201.97±1/1 | 182.6±0.62 | 167.99±0/66 | 155.43±0/49 |


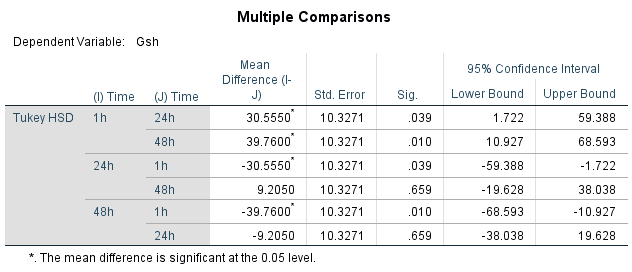

Supplement: S4 Table — (DOCX) [file pone.0290947.s004.docx]

S5 Table. Catalase in different time pointes

| **Time**  **Concentration** | **1h** | **12h** | **24h** | **48h** |
| --- | --- | --- | --- | --- |
| **50 μg/ml** | 4.776  ±0/17 | 4.826 ± 0.42 | 4.873  ±0/16 | 4.284  ±1/76 |
| **25 μg/ml** | 5.411  ±2/24 | 5.141 ± 0.26 | 5.08  ±0/99 | 4.654  ±1/1 |
| **12 μg/ml** | 5.684  ±0/55 | 5.412 ± 0.19 | 5.136  ±0/54 | 5.096  ±0/78 |
| **6 μg/ml** | 6.193  ±0/23 | 4.811 ± 1.11 | 5.371  ±1/9 | 5.192  ±1/2 |


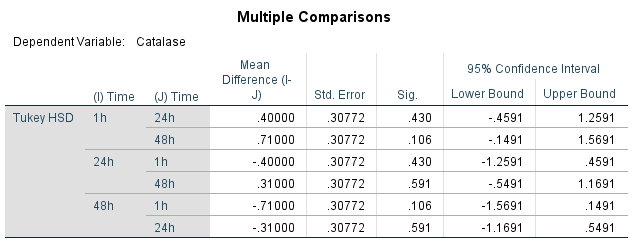

Supplement: S5 Table — (DOCX) [file pone.0290947.s005.docx]

S6 Table. SOD in different time pointes

| **Time**  **Concentration** | **1h** | **12h** | **24h** | **48h** |
| --- | --- | --- | --- | --- |
| **50 μg/ml** | 134.51±0/67 | 133.42± 0.34 | 131.51±0/29 | 129.62±0/79 |
| **25 μg/ml** | 139.30±0/54 | 138.21 ± 0.29 | 135.07±1/3 | 134.62±0/51 |
| **12 μg/ml** | 145.33±1/5 | 145.53 ± 0.87 | 146.32±1/24 | 144.64±1/13 |
| **6 μg/ml** | 147.23±0/33 | 148.63 ± 0.36 | 147.45±0/95 | 144.11±0/61 |


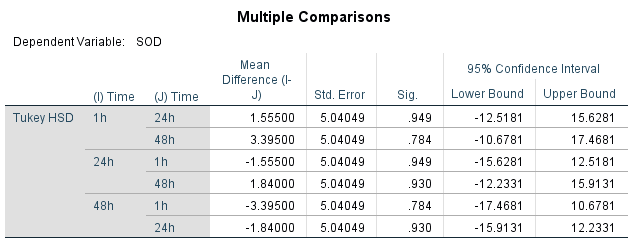

Supplement: S6 Table — (DOCX) [file pone.0290947.s006.docx]
