# Supplementary material for: In vitro and in silico scolicidal effect of sanguinarine on the hydatid cyst protoscoleces
Source: PLoS One. 2023 Oct 25;18(10):e0290947. doi: 10.1371/journal.pone.0290947 (PMC10599545; doi:10.1371/journal.pone.0290947)
Supplement: S7 Table — (DOCX) [file pone.0290947.s007.docx]

S7 Table. GPX in different time pointes

| **Time**  **concentration** | **1h** | **12h** | **24h** | **48h** |
| --- | --- | --- | --- | --- |
| **50 μg/ml** | 5.814 ±1/7 | 4.912 ±0.87 | 4.1558 ±0/5 | 3.1168 ±0/98 |
| **25 μg/ml** | 7.272 ±1/55 | 6.12 ± 1.03 | 6.233 ±1.5 | 3.7363 ±1/25 |
| **12 μg/ml** | 10.289 ±0/53 | 8.711 ± 0.32 | 7.2727 ±0/98 | 5.8142 ±0/19 |
| **6 μg/ml** | 15.584 ±1/3 | 12.6 ± 0.96 | 9.870 ±0/54 | 7.7922 ±0/76 |
